# Incidence and risk factors of neonatal hypoglycemia after ritodrine therapy in premature labor: a retrospective cohort study

**DOI:** 10.1186/s40780-019-0137-3

**Published:** 2019-04-16

**Authors:** Shoko Shimokawa, Akiko Sakata, Yukio Suga, Kazuya Isoda, Shingo Itai, Katsuhiko Nagase, Tsutomu Shimada, Yoshimichi Sai

**Affiliations:** 1 Department of Clinical Pharmacokinetics, Graduate School of Medical Sciences, Kanazawa University, 13-1 Takara-machi, Kanazawa, 920-8641 Japan; 2 Department of Hospital Pharmacy, University Hospital, Kanazawa University, 13-1 Takara-machi, Kanazawa, 920-8641 Japan; 3 Department of Clinical Drug Informatics, Faculty of Pharmacy, Institute of Medical, Pharmaceutical and Health Sciences, Kanazawa University, Kakuma-machi, Kanazawa, 920-1192 Japan; 4 Innovative Clinical Research Center, University Hospital, Kanazawa University, 13-1 Takara-machi, Kanazawa, 920-8641 Japan

**Keywords:** Neonatal hypoglycemia, Ritodrine hydrochloride, Tocolytics, Premature labor

## Abstract

**Background:**

Ritodrine hydrochloride (RD), a β2-adrenergic agonist, is widely used as a tocolytic medication to suppress premature labor, but can cause neonatal hypoglycemia, a potentially severe side effect. We examined the incidence and risk factors of neonatal hypoglycemia following maternal intravenous administration of RD.

**Methods:**

This was a retrospective study of neonates, who had birth weight of ≥2000 g and were delivered at 36 weeks gestation or later in Kanazawa University Hospital from August 2013 to July 2016. We defined neonatal hypoglycemia as blood glucose level < 50 mg/dL. Neonates who were delivered without maternal intravenous RD or who were delivered 8 days or more after stopping maternal RD or who received oral RD were defined as the RD non-administration group, while those delivered within 7 days after stopping maternal RD were defined as the RD intravenous administration group. We examined the incidence and risk factors of RD-induced neonatal hypoglycemia by comparing these two groups.

**Results:**

We enrolled 603 neonates in this study; 504 (83.6%) showed no neonatal hypoglycemia, while 99 (16.4%) exhibited neonatal hypoglycemia. The incidence of neonatal hypoglycemia was significantly higher (61.7%; 58/94) in the RD intravenous administration group than in the RD non-administration group (8.1%; 41/509) (*p* < 0.001). Binomial logistic regression analysis in the RD intravenous administration group showed that maternal age over 35 years (AOR: 3.385; 95% CI, 1.082–10.588, *p* = 0.036) and the interval to delivery from stopping intravenous administration of RD (AOR: 0.974; 95% CI, 0.953–0.996, *p* = 0.020) were independent factors associated with neonatal hypoglycemia. The cut-off value of the interval to predict the incidence of neonatal hypoglycemia was about 6 h (sensitivity 82.8%, specificity 63.9%).

**Conclusions:**

The incidence of neonatal hypoglycemia was significantly increased by maternal intravenous administration of RD. We newly identified maternal age (over 35 years) and the interval to delivery from stopping intravenous administration of RD (within 6 h) as independent risk factors for neonatal hypoglycemia following maternal intravenous administration of RD. In cases with these risk factors, careful blood glucose monitoring is recommended for early detection and treatment of neonatal hypoglycemia.

## Introduction

Ritodrine hydrochloride (RD), a β2-adrenergic agonist, is widely used as a tocolytic medication to suppress premature labor [1]. However, it can cause severe and diverse side effects, including pulmonary edema, agranulocytosis, and rhabdomyolysis, and the US Food and Drug Administration (FDA) and the European Medicines Agency (EMA) have recommended discontinuation of oral administration of RD [2–6]. Moreover, there is evidence that the efficacy of tocolytic medication is limited to 48 h [7]. Nevertheless, there is currently no restriction on administration of RD in Japan, and either oral or intravenous long-term administration of RD may be used at the physician’s discretion to prevent recurrence of premature labor, even after uterine contraction is suppressed. However, a recent survey of the use of RD in Japan showed that about 20% of patients (mothers) given RD encountered adverse events such as liver dysfunction and rhabdomyolysis [8]*.* Furthermore, there are some case reports in Japan describing heart failure in neonates delivered following maternal administration of RD for more than 2 weeks [9]. In view of these reports, a survey of the effects and side effects of magnesium sulfate and RD on neonates delivered between 32 and 36 weeks of gestation is being conducted by the Japan Society of Perinatal and Neonatal Medicine. The results of these survey will provide the basis for a reconsideration of the appropriate therapy in Japan [10, 11].

Neonatal hypoglycemia is also recognized as one of the side effects of RD, and can have severe neurological sequelae, such as developmental disturbance due to brain damage, convulsions, and cerebral palsy. Previous studies have found that low birth weight, preterm birth, multiple pregnancy, congenital metabolic disorder, maternal diabetes, and maternal drug treatment (β2-adrenergic agonist, sodium valproate, etc.) are risk factors for neonatal hypoglycemia [12, 13]. In particular, the incidence of neonatal hypoglycemia was increased in neonates delivered by mothers who had received isoxsuprine, while increased blood insulin and decreased blood glucose were found in neonates delivered by mothers who had received fenoterol or terbutaline [14, 15]. Although RD shows high selectivity for uterine muscle compared with other β2-adrenergic agonists, and is used as the first-line tocolytic medication in Japan [16], few studies have examined the association between maternal administration of RD and neonatal hypoglycemia.

Therefore, the aim of this study was to examine the incidence of neonatal hypoglycemia following maternal administration of RD, and to identify the risk factors.

## Method

### Study design

This retrospective study cohort recruited neonates who were delivered after 36 weeks of gestation in Kanazawa University Hospital from August 2013 to July 2016. Exclusion criteria were birth weight < 2000 g, since glucose is administered immediately after birth in such cases, and the presence of underlying disease. Since week number is commonly used to define stages of pregnancy and in discussing the condition of the mother and fetus during pregnancy in clinical practice, we adopted a time period of one week to divide the two groups in this study: neonates who were delivered within 7 days after stopping maternal intravenous RD were defined as the RD intravenous administration group, and those who delivered by mothers who did not receive intravenous RD or who were delivered 8 days or more after stopping maternal RD or who received oral RD were defined as the RD non-administration group. The reason for including patients who received oral RD in the latter group was that the dose of oral RD is small and oral RD was excluded as a risk factor for neonatal hypoglycemia in this study. We also divided the cohort in terms of oral RD and magnesium sulfate in order to evaluate them as risk factors in the same manner as the RD intravenous administration group. Neonatal hypoglycemia is defined as a blood glucose level less than 50 mg/dL in the department of Obstetrics and Gynecology at Kanazawa University Hospital, and neonatal hypoglycemia is treated with oral lactation or intravenous administration of glucose.

To identify risk factors of neonatal hypoglycemia, we collected and examined the following data: maternal age, maternal height, pre-pregnancy body mass index (BMI), maternal weight gain, presence or absent of maternal diabetes, maternal underlying disease, single or multiple pregnancy (in this study, only twins; hereafter referred to as twin births), birth weight, gestational age, neonatal blood glucose level, usage of other preterm labor drugs, maternal intravenous administration period of RD, the final administration dose rate of RD, and the interval to delivery from stopping maternal administration of RD. The schedule of neonatal blood glucose level measurement at our hospital depends upon gestational age, birth weight, and intravenous administration or non-administration of RD, as shown in Table 1.

**Table 1:** Neonatal blood glucose monitoring

Gestational age/Birth weight	Intravenous administration of RD	After birth	1 h after birth	3 h after birth
< 37 weeks or < 2500 g	+	〇	〇	〇
	–			
37 weeks ≤ and 2500 g ≤	+		〇	〇
	–			〇

*RD* Ritodrine hydrochloride

Neonatal blood glucose monitoring was carried out at the times indicated by circles in the table, according to the classification by gestational age, birth weight, and intravenous administration or non-administration of RD

This study was approved by the ethics committee of Kanazawa University of Health (No.2016–146). All analyses were performed using anonymized date.

### Statistical analysis

To examine the risk factors of neonatal hypoglycemia in all 603 cases, we selected patients’ characteristics previously reported to be related to the incidence of neonatal hypoglycemia and compared their occurrence rates in the no neonatal hypoglycemia group with those in the neonatal hypoglycemia group using the Mann - Whitney *U* - test or Fisher’s exact test. Binomial logistic regression analysis was also performed to eliminate confounding factors.

To examine the risk factors of neonatal hypoglycemia in the RD intravenous administration group, a comparative study of patients’ characteristics in the two groups was similarly performed, and binomial logistic regression analysis was also performed. The analysis included parameters for which significant differences were found in the comparative study. In the analysis, maternal age was categorized as less or more than 35, because this is defined as the age of elderly primigravida, according to the Japanese Society of Obstetrics and Gynecology. A continuous variable that showed statistical significance was analyzed in terms of the receiver operating characteristic (ROC) curve. A *p* value less than 0.05 was considered significant.

All statistical analysis was performed using GraphPad Prism 6 J (MDF Co., Ltd., Tokyo) or SPSS ver. 24.0 J (IBM Japan Ltd., Tokyo).

## Results

### Patients’ characteristics

Table 2 shows the clinical characteristics of the 603 neonates. Median birth weight was 2904 g and number of twins was 54. Maternal underlying diseases were detected in about half of the mothers. RD was administered orally in 85 and intravenously in 94 patients, and magnesium sulfate was administered in 17 patients. In all cases, drug dosage and administration were carried out in compliance with the corresponding package insert provided in Japan.

**Table 2:** Patients’ characteristics

Cases (n)	603
Maternal age (years)	33 (17 – 47)
Maternal height (cm)	159.0 (140.0 – 177.0)
Pre-pregnancy BMI (kg/m^2^)	20.3 (14.2 – 38.1)
Maternal weight gain (kg)	9.8 (−10.3 – 22.0)
Birth weight (g)	2904 (2002 – 4336)
Gestational age (wk/day)	38/3 (36/0 – 41/6)
Twin birth (*n*)	54 (9.0)
Maternal underlying diseases (*n*)	261 (43.3)
Maternal diabetes (*n*)	26 (4.3)
Oral administration of RD (*n*)	85 (14.1)
Intravenous administration of RD (*n*)	94 (15.6)
Administration of magnesium sulfate (*n*)	17 (2.8)

Data are expressed as median (min-max) or number (%)

*BMI* Body mass index, *RD* Ritodrine hydrochloride

### Risk factors of neonatal hypoglycemia in all cases

We compared patients’ characteristics previously reported to be related to the incidence of neonatal hypoglycemia in the no neonatal hypoglycemia group with those in the neonatal hypoglycemia group. Significant differences were found in gestational age, birth weight less than 2500 g, twin birth, and intravenous administration of RD. Binomial logistic regression analysis was further performed, and showed that gestational age (AOR: 0.516; 95% CI, 0.366–0.726, *P* < 0.001), birth weight less than 2500 g (AOR: 3.484; 95% CI, 1.856–6.540, *P* < 0.001), and intravenous administration of RD (AOR: 6.595; 95% CI, 3.307–13.153, P < 0.001) were independent factors associated with neonatal hypoglycemia (Table 3). There was no multicollinearity between gestational age and birth weight less than 2500 g.

**Table 3:** Analysis of the risk factors of neonatal hypoglycemia in all cases (*n* = 603)

	No Neonatal Hypoglycemia	Neonatal Hypoglycemia	*p* value	AOR	95%CI	*p* value
Case (*n*)	504 (83.6)	99 (16.4)				
Gestational age (wk/day)	38/5 (36/0 – 41/6)	37/1 (36/0 – 41/2)	< 0.001	0.516	0.366–0.726	< 0.001
Birth weight < 2500 g (*n*)	57 (11.3)	47 (47.5)	< 0.001	3.484	1.856–6.540	< 0.001
Twin birth (*n*)	24 (4.8)	30 (30.3)	< 0.001	1.645	0.772–3.505	0.197
Maternal diabetes (*n*)	21 (4.2)	5 (5.1)	0.598	1.272	0.358–4.521	0.710
Oral administration of RD (*n*)	68 (13.5)	17 (17.2)	0.344	0.806	0.393–1.654	0.557
Intravenous administration of RD (*n*)	36 (7.1)	58 (58.6)	< 0.001	6.595	3.307–13.153	< 0.001

Data are expressed as median (min-max) or number (%)

*RD* Ritodrine hydrochloride, *AOR* Adjusted odds ratio, *CI* Confidence interval

### Comparison of the incidence of neonatal hypoglycemia in the RD intravenous administration group and RD non-administration group

**Fig. 1:**
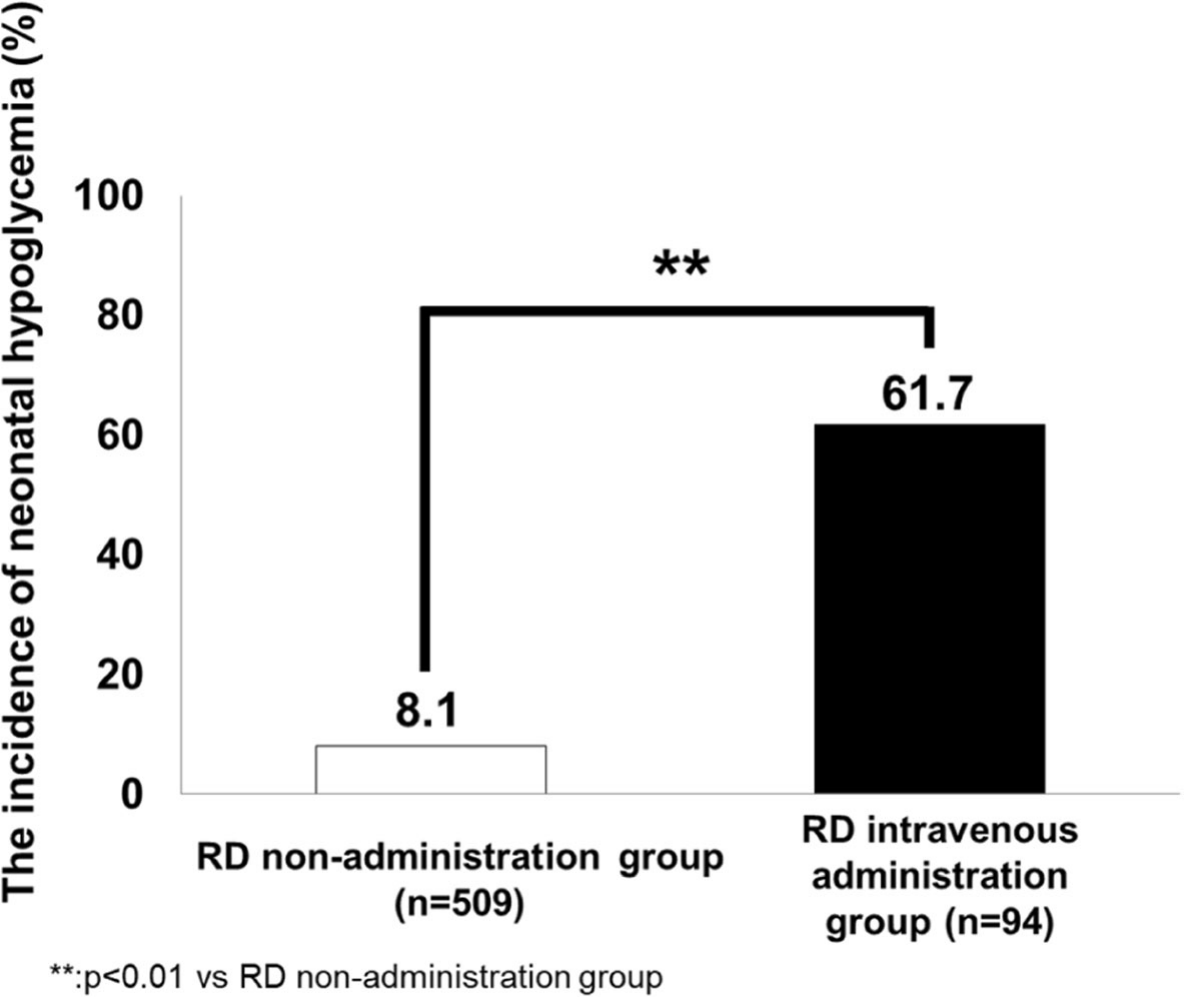
Incidences of neonatal hypoglycemia in the RD non-administration group and RD intravenous administration group

The incidence of neonatal hypoglycemia in the RD intravenous administration group (61.7%; 58/94) was significantly higher than that in the RD non-administration group (8.1%; 41/509) (*p* < 0.001) (Fig. 1).

### Risk factors of neonatal hypoglycemia in the RD intravenous administration group

Significant differences between the RD intravenous administration group and the RD non-administration group were found in maternal age over 35, birth weight less than 2500 g, twin birth, administration dose rate at the end of intravenous administration of RD, and interval to delivery from stopping intravenous administration of RD. In order to eliminate confounding factors, binomial logistic regression analysis including the above five factors was further performed. Maternal age over 35 (AOR: 3.385; 95% CI, 1.082–10.588, *P* = 0.036) and the interval from stopping intravenous administration of RD to delivery (AOR: 0.974; 95% CI, 0.953–0.996, *P* = 0.020) were identified as independent factors associated with neonatal hypoglycemia (Table 4).

**Table 4:** Analysis of the risk factors of neonatal hypoglycemia in the RD intravenous administration group

	No Neonatal Hypoglycemia	Neonatal Hypoglycemia	*P* value	AOR	95%CI	*P* value
Cases (*n*)	36 (38.3)	58 (61.7)				
Maternal age over 35 (*n*)	7 (19.4)	24 (41.4)	0.042	3.385	1.082–10.588	0.036
Maternal height (cm)	158.5 (140.0 – 169.0)	158.0 (146.0 – 174.0)	0.703			
Pre-pregnancy BMI (kg/m^2^)	20.4 (15.3 – 26.6)	20.3 (17.0 – 33.8)	0.876			
Maternal weight gain (kg)	8.0 (1.5 – 18.0)	9.0 (3.0 – 17.5)	0.117			
Gestational age (wk/day)	37 /1 (36/0 – 38/4)	37/0 (36/0 – 37/6)	0.598			
Birth weight < 2500 g (*n*)	7 (19.4)	26 (44.8)	0.015	2.479	0.774–7.942	0.126
Twin birth (*n*)	6 (16.7)	25 (43.1)	0.012	2.417	0.743–7.863	0.143
Administration of magnesium sulfate (*n*)	4 (11.1)	11 (19.0)	0.393			
Administration length (day)	43.5 (0.04 – 134.0)	38.5 (6.0 – 147.0)	0.694			
Final administration dose rate (μg/min)	65.0 (33.0 – 192.3)	93.3 (32.1 – 192.3)	0.048	0.999	0.987–1.011	0.878
Interval (hr)	12.5 (0.0 – 154.0)	1.3 (0.0 – 167.9)	< 0.001	0.974	0.953–0.996	0.020

Data are expressed as median (min-max) or number (%). The final administration dose rate is that at the time of stopping maternal intravenous administration of RD. The interval is the time period from stopping maternal intravenous administration of RD to delivery

RD: ritodrine hydrochloride, AOR adjusted odds ratio, CI confidence interval

### Relationship between the interval to delivery from stopping intravenous administration of RD and the risk of neonatal hypoglycemia

**Fig. 2:**
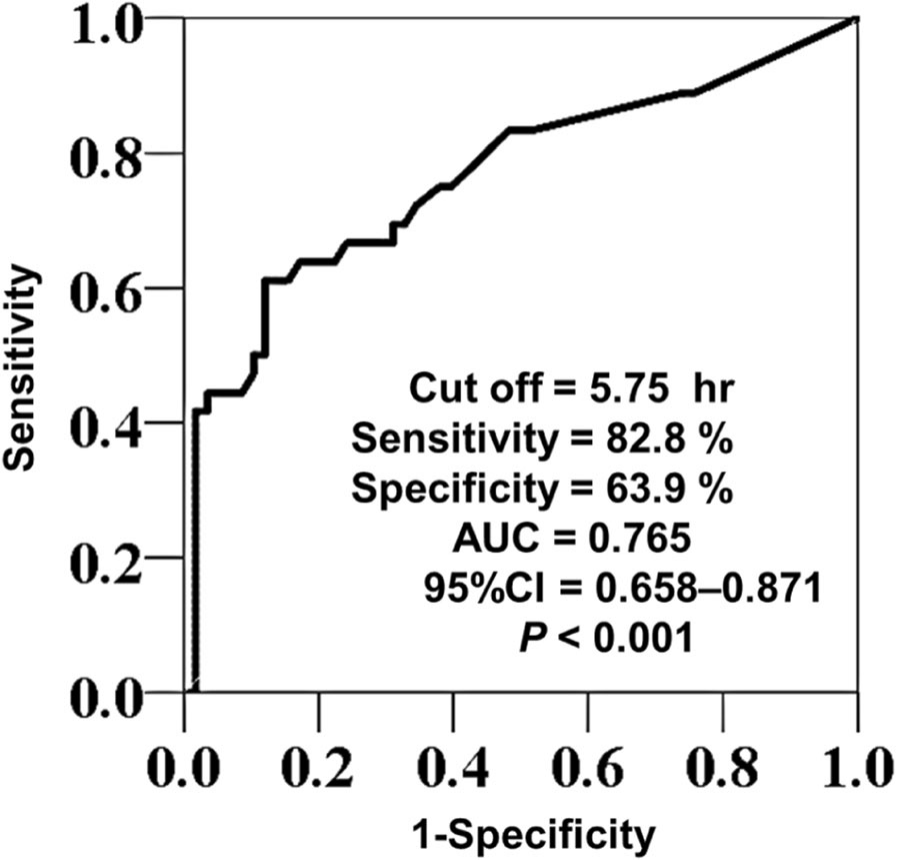
Receiver operating curve for predicting the incidence of neonatal hypoglycemia in relation to the interval. The interval is the time period from stopping maternal intravenous administration of RD to delivery. RD: ritodrine hydrochloride, AUC: area under the curve

We examined the ROC curve for the relationship between the interval to delivery from stopping intravenous administration of RD and the risk of neonatal hypoglycemia. The area under the curve (AUC) was 0.765 (95% CI, 0.658–0.871, *P* < 0.001), and the cut-off value was calculated to be 5.75 h. The cut-off value provided a sensitivity of 82.8%, specificity of 63.9%, positive predictive value of 78.7%, negative predictive value of 69.7%, and accuracy of 75.5% (Fig. 2).

## Discussion

Two mechanisms are reported to be involved in the development of neonatal hypoglycemia. One is insufficient glycogenesis due to insufficiency of glycogen, due to immaturity of neonates (e.g., preterm birth or birth weight less than 2500 g). The other is increased glucose consumption including hyperinsulinemia, and is caused by maternal diabetes and maternal drug treatment (β2-adrenergic agonist, sodium valproate, etc.) [12]*.* In the case of maternal administration of β2-adrenergic agonist, gluconeogenesis is promoted in the maternal liver, and the maternal blood glucose level rises, resulting in neonatal hyperinsulinemia, similar to the case of neonates in maternal diabetes [15]. In addition, previous studies have reported that RD easily passed through the blood-placenta barrier. Van Lierde et al. reported that the concentration of RD on the fetal side reached about 20 to 30% of that on the maternal side. Gross et al. also reported that the RD concentration on the fetal side was higher than that on the maternal side. Fetal RD could lead to continuous pancreatic β cell stimulation from the fetal stage through the neonatal period, causing insulin hypersecretion and neonatal hypoglycemia [17–19]. All these results are consistent with our findings that earlier gestational age, birth weight less than 2500 g, and intravenous administration of RD are independent risk factors of neonatal hypoglycemia. On the other hand, although maternal diabetes and oral administration of RD were reported to play roles in neonatal hypoglycemia, we found no significant effects in this study. Possible reasons for this include the small number of cases with maternal diabetes (26 cases, 4.3%) in our study, and the use of lower dosages of oral RD in Japan compared with those previously used in Europe and the US [7, 20].

In this work, comparison of the two groups indicated that the incidence of neonatal hypoglycemia is increased about 8 times by intravenous administration of RD. Logistic regression analysis in RD intravenous administration group revealed that maternal age over 35 and the interval to delivery from stopping intravenous administration of RD were independent risk factors of neonatal hypoglycemia. Generally maternal age over 35 has been defined as the age of elderly primigravida, and reported as a risk factor for delivery; also, greater maternal age is associated with increased probability of infertility and increased rates of miscarriage, complications during pregnancy, and congenital anomaly [21, 22]. However, this is the first report to indicate that the maternal age over 35 years is a risk factor of neonatal hypoglycemia following maternal intravenous administration of RD. As for the relationship between the interval to delivery from stopping intravenous administration on RD and the risk of neonatal hypoglycemia, Motai et al. reported that the incidence of neonatal hypoglycemia increased as the interval to delivery from stopping intravenous RD administration was shorter [23]*.* We examined the cut-off value for the risk of neonatal hypoglycemia using ROC curve analysis. The AUC was 0.765, indicating moderate predictive ability. The cut-off value was calculated to be 5.75 h. Overall, these findings indicate that the incidence of neonatal hypoglycemia can be reduced by stopping maternal intravenous administration of RD at least 6 h before delivery.

As regards limitations of our study, it was conducted at a single center, and was a retrospective cohort study. Although maternal age, maternal height, pre-pregnancy BMI, maternal weight gain and gestational age showed similar values to those reported in previous studies [24, 25], the twin birth rate was as high as 9.0% in this study cohort, while it is about 2.0% in the general population [26]. This could be a source of bias in our study population. In order to confirm these results in the general population, and to eliminate various possible biases, a multicenter and cohort study would be needed. Moreover, since there is no clear rationale for choosing the time point to divide patients into RD and non-RD groups, further consideration of this point may be necessary.

Despite the present findings, intravenous administration of RD is considered to be extremely important to prevent premature labor in Japan. Therefore, we recommend careful monitoring of blood glucose level in neonates delivered by mothers having these risk factors, in order to ensure rapid treatment of neonatal hypoglycemia. In fact, no sequelae due to neonatal hypoglycemia were observed in our study, suggesting that the schedule of blood glucose monitoring according to the patients’ background currently employed in our hospital is suitable for preventing sequelae due to neonatal hypoglycemia.

## Conclusion

The incidence of neonatal hypoglycemia was significantly increased by intravenous administration of RD in our single-center, retrospective cohort study. We identified maternal age (over 35 years) and interval to delivery from stopping intravenous administration of RD of less than 6 h as independent risk factors of neonatal hypoglycemia following maternal intravenous administration of RD. We recommend careful blood glucose monitoring in neonates whose mothers have these risk factors, in order to ensure early detection and treatment of neonatal hypoglycemia.

## Abbreviations

AOR: adjusted odds ratio
AUC: area under the curve
BMI: body mass index
CI: confidence interval
RD: ritodrine hydrochloride

## References

[1] Takagi K, Satoh T (2009). Is long-term tocolysis effective for threatened premature labor?. J Int Med Res.

[2] Verriello L, D'Amico D, Pauletto G, Gigli GL, Bergonzi P (2009). Rhabdomyolysis caused by tocolytic therapy with ritodrine hydrochloride. Neuromuscul Disord.

[3] Gezginç K, Gül M, Karatayli R, Cander B, Kanat F (2008). Noncardiogenic pulmonary edema due to ritodrine usage in preterm labor. Taiwan J Obstet Gynecol.

[4] Yasuda R, Makino Y, Matsuda Y, Kawamichi Y, Matsui H (2012). Agranulocytosis associated with intravenous ritodrine hydrochloride therapy: two case reports by different mechanisms. J Obstet Gynaecol Res.

[5] Haas DM, Benjamin T, Sawyer R, Quinney SK (2014). Short-term tocolytics for preterm delivery - current perspectives. Int J Women's Health.

[6] EUROPEAN MEDICINES AGENCY: PRAC recommends restricted use of short-acting beta-agonists in obstetric indications. http://www.ema.europa.eu/docs/en_GB/document_library/Referrals_document/Short-acting_beta-agonists/Recommendation_provided_by_Pharmacovigilance_Risk_Assessment_Committee/WC500148669.pdf. Accessed 20 Dec 2017.

[7] Neilson JP, West HM, Dowswell T. Betamimetics for inhibiting preterm labour. Cochrane Database Syst Rev. 2014;doi:10.1002/14651858.CD004352.pub3. 10.1002/14651858.CD004352.pub3 PMC10603219 24500892

[8] Otsuki K (2016). Survey report on the actual use of ritodrine hydrochloride and side effects. Year Book of Japan Society of Perinatal and Neonatal Medicine.

[9] Pharmaceutical and Food Safety Bureau, Ministry of Health, Labour and Welfare: Pharmaceuticals and Medical Devices Safety Information No.285. http://www.pmda.go.jp/files/000152990.pdf. Accessed 20 Dec 2017.

[10] Nakamura M, Hasegawa J, Arakaki T, Hamada S, Takita H, Oba T (2016). Comparison of perinatal outcomes between long-term and short-term use of tocolytic agent: a historical cohort study in a single perinatal hospital. J Obstet Gynaecol Res.

[11] Yoneda S, Yoneda N, Fukuta K, Shima T, Nakashima A, Shiozaki A (2018). In which preterm labor-patients is intravenous maintenance tocolysis effective?. J Obstet Gynaecol Res.

[12] Williams AF (1997). Hypoglycaemia of the newborn: a review. Bull World Health Organ.

[13] Adamkin DH (2011). Postnatal glucose homeostasis in late-preterm and term infants. Pediatrics..

[14] Brazy JE, Pupkin MJ (1979). Effects of maternal isoxsuprine administration on preterm infants. J Pediatr.

[15] Epstein MF, Nicholls E, Stubblefield PG (1979). Neonatal hypoglycemia after beta-sympathomimetic tocolytic therapy. J Pediatr.

[16] Takagi K (2011). Tocolytics for Management of Preterm Labor: use of β agonists as the first line Tocolytic agents. Acta obstetrica et gynaecologica Japonica..

[17] Procianoy RS, Pinheiro CE (1982). Neonatal hyperinsulinism after short-term maternal beta sympathomimetic therapy. J Pediatr.

[18] van Lierde M, Thomas K (1982). Ritodrine concentrations in maternal and fetal serum and amniotic fluid. J Perinat Med.

[19] Gross TL, Kuhnert BR, Kuhnert PM, Rosen MG, Kazzi NJ (1985). Maternal and fetal plasma concentrations of ritodrine. Obstet Gynecol.

[20] Dodd JM, Crowther CA, Middleton P. Oral betamimetics for maintenance therapy after threatened preterm labour. Cochrane Database Syst Rev. 2012. 10.1002/14651858.CD003927.pub3. 10.1002/14651858.CD003927.pub3 PMC10964130 23235600

[21] Koike K (2000). Problems of infertility in aging women: the effects of aging on the ovarian function. Acta obstetrica et gynaecologica Japonica.

[22] Ueda K (2000). Problems of infertility in aging women: abortion. Acta obstetrica et gynaecologica Japonica..

[23] Motai K, Nagata S, Watanabe T (1989). Hypoglycemia of the neonate after maternal treatment of premature labor with ritodrine hydrochloride. Matsuyama R. C. Hosp J Med.

[24] Takimoto H, Sugiyama T, Fukuoka H, Kato N, Yoshiike N (2006). Maternal weight gain ranges for optimal fetal growth in Japanese women. Int J Gynecol Obstet.

[25] Terada M, Matsuda Y, Ogawa M, Matsui H, Satoh S (2013). Effects of maternal factors on birth weight in Japan. J Pregnancy.

[26] Ministry of Healthy Labor and Welfare: Birth statistics. http://www.mhlw.go.jp/toukei/saikin/hw/jinkou/tokusyu/syussyo06/index.html. Accessed 20 Dec 2017.

